# Comment on: “Use of thermographic imaging for the evaluation of erectile dysfunction and Peyronie’s disease”

**DOI:** 10.1038/s41443-024-00952-0

**Published:** 2024-07-18

**Authors:** Raevti Bole

**Affiliations:** Glickman Urological Institute, Cleveland Clinic Foundation, Cleveland, OH USA

**Keywords:** Erectile dysfunction, Sexual dysfunction

Penile duplex Doppler ultrasound (PDDU) is a well-established tool for the evaluation of erectile dysfunction (ED). Though hemodynamic assessment is not required for the workup or treatment of the index ED patient, PDDU proves useful to distinguish between vascular and nonvascular ED. In this paper, the authors present a proof-of-concept study to explore the use of an even more minimally invasive measure of erectile function- thermographic imaging [1].

The study authors hypothesize that thermographic evaluation of pharmacologically induced erection will correlate to established measures of hemodynamics by PDDU. This is a concept borne out of prior work on genital blood flow changes during subjective sexual arousal [2]. Thermography testing is easier to perform and less variable than PDDU, which requires operator expertise. The authors found that in a cohort of patients with either ED or Peyronie’s disease (PD), there was a significant correlation between change in temperature and change in peak systolic velocity (PSV), although no change in end-diastolic velocity (EDV) or resistive index (RI). The findings are of interest, though it remains to be seen whether this principle applies to healthy patients without ED/PD and whether thermography can distinguish between normal versus diminished arterial flow.

A potential confounder of the study findings is the inclusion of PD patients in the cohort, as these patients typically experience a higher rate of veno-occlusive dysfunction. This may have affected the analysis of EDV, RI, and temperature change correlation. In order for thermographic imaging to gain clinical utility, future studies would need to demonstrate reliable thermographic parameters that correlate to normal hemodynamics, arterial insufficiency, and also veno-occlusive dysfunction.

The authors also report a non-significant difference in plaque temperature of PD patients compared with the remainder of the penis. The reason for this is unclear, though plaque density may affect heat conductivity as measured by thermography. Skin thickness has been shown to affect thermographic imaging in studies of lower extremity wounds and may provide a possible mechanism here [3]. Further studies on the correlation of plaque temperature to variables such as plaque size and grade of calcification would be relevant.

Overall, while these preliminary data do not seek to prove the superiority of thermographic imaging over PDDU, they do provide a basis for further study of non-invasive techniques for accurate and reliable evaluation of ED and PD.

## References

[1] Crisostomo-Wynne T, Hertz A, Maloney T, Walter J, Caras R. Use of thermographic imaging for the evaluation of erectile dysfunction and Peyronie’s disease. Int J Impot Res. 2024. 10.1038/s41443-024-00950-2 10.1038/s41443-024-00950-2 39004622

[2] Kukkonen TM, Binik YM, Amsel R, Carrier S. An evaluation of the validity of thermography as a physiological measure of sexual arousal in a non-university adult sample. Arch Sex Behav. 2010;39:861–73. 10.1007/s10508-009-9496-4 19387817 10.1007/s10508-009-9496-4

[3] Sadrzadeh-Afsharazar F, Raizman R, Saiko G. Utility of thermographic imaging for callus identification in wound and foot care. Sensors. 2023;23:9376 10.3390/s23239376 38067749 10.3390/s23239376 PMC10708640

