# Correction: Comment on: “Use of thermographic imaging for the evaluation of erectile dysfunction and Peyronie’s disease”

**DOI:** 10.1038/s41443-024-00958-8

**Published:** 2024-08-23

**Authors:** Raevti Bole

**Affiliations:** Glickman Urological Institute, Cleveland Clinic Foundation, Cleveland, OH USA

**Keywords:** Erectile dysfunction, Sexual dysfunction

Correction to: *International Journal of Impotence Research* 10.1038/s41443-024-00952-0, published online 18 July 2024

In this article the title was incorrectly given as ‘Comment on the article IJIR-08-2023-242R - use of thermographic imaging for the evaluation of erectile dysfunction and Peyronie’s disease’

but should have been

Comment on: “Use of thermographic imaging for the evaluation of erectile dysfunction and Peyronie’s disease”

The link in Reference 1 has been corrected to:

10.1038/s41443-024-00950-2

